# Woman: An Oration Delivered before the American Academy of Dental Science, Boston, Nov. 7, 1883

**Published:** 1884-03

**Authors:** N. W. Kingsley

**Affiliations:** New York


### WOMAN.

AN ORATION DELIVERED BEFORE THE AMERICAN ACADEMY OF DENTAL SCIENCE, BOSTON, NOVEMBER 7, 1883.

BY N. W. KINGSLEY, D. D. S., NEW YORK.

Two days' journey from Boston, half a century ago, the traveler would have found among the Green Mountains of Vermont a charming little village, situated upon a plateau, with a background of mountains, and a valley bounding it on three sides.

Across the valley other mountain peaks arose in every direction, breaking and beautifying the horizon line around the entire circle.

The evening sunlight, and the morning sun as well, developed a landscape which for picturesque beauty can nowhere be excelled. The meadows and the pastures, the groves of pine, of beech and of maple, the quiet river, and the laughing, dashing brooks, the little cascades and the larger waterfalls, formed a scene which in certain conditions of the atmosphere became surpassingly enchanting.

In the spring-time, when the river was swollen by freshets, the roar of a cataract higher than Niagara was heard for miles, and the meadows became a vast inland lake. No gorgeous sunsets ever exalted an enthusiast more than those which at times illuminated the ravines and faded away upon the mountain peaks.

No grander or more startling peals of thunder, reverberating from crag to crag, ever terrified the timid more than at times broke over the stillness of that secluded spot. A peaceful, virtuous and happy people made up that community, almost Arcadian in its simplicity, but withal a refined, educated and cultured people. Even in its district schools the sciences and the classics were taught by students of Harvard and Dartmouth, and in the village was a well-selected library of several thousand volumes, suited to all ages and all tastes.

On the Sabbath in that day every farm-house in the entire community was almost deserted during the hours of church service, for the people were not only a religious people, but they still retained the Puritanical forms. The Sabbath began on Saturday at sunset, and the boys felt that they had committed a sin if they continued ball-playing into the twilight.

On a Sunday at that time might have been seen in the village church, on the hill, he who is now the most noted skeptic living, listening to his own father's exposition of the brimstone doctrines which the son now ridicules.

Memory calls up a lad of that day who devoured with avidity all the literature specially adapted to youth which the library then contained. The volumes were few, and they were chiefly devoted to descriptions of life in Boston; but the style was charming, and the incidents related so fascinating that they were almost learned by heart.

It was the lad's first knowledge of city life, and to him Boston was the most attractive and most delightful place on earth. In his imagination it was the center and abode of all learning and all art. It was the home of polite society, and an aggregation of intellect and culture. There was a glamour over everything relating to Boston, in that youthful mind, which has never been obliterated nor superseded; and to-day, as a half century is drawing to a close, he stands here, your chosen orator upon this occasion, and he thinks of Boston as the ideal city of the modern world.

With such a respect for the intellectuality of a Boston audience, is it any wonder that I should have hesitated in accepting such an appointment, or would it be regarded as a matter of surprise that I should be at a loss for a theme?

It is a laudable ambition in any speaker which prompts him to avoid well-trodden paths, and seek to develop new and unexplored fields. It is an assurance bordering almost upon conceit with which a speaker comes voluntarily before an audience, and addresses them upon a subject which has become threadbare with repetition. I have endeavored, heretofore, to avoid all such absurdities, and whether in speaking or writing, have generally succeeded in introducing topics that were at least comparatively new; and yet I am about to address you upon a topic more trite and more hackneyed than any other; one which has been the theme of writers from the earliest ages, and one which, at one period or another, if not at the present moment, has concerned each man in my audience more than his immortal welfare.

Our topic to-day is "WOMAN."

Is there any other subject, in all the ages, that would not have been long since exhausted and relegated to the domain of ancient history? Yet, like the kaleidoscope, every turn brings a new revelation, and my only justification upon this occasion is that I have something to say to a gathering of dentists which is specially applicable to them, and which, so far as I know, has not heretofore been said.

What is woman? and what her characteristics and capabilities?

If an intelligent being from another planet had no other means of obtaining a knowledge of woman than by reading our literature, what opinion would he form, and to what conclusion would he come?

Is she a goddess, or a demon? angelic or satanic?

In classic and in modern literature, sages and poets have made her the object of many satires, of much praise, and of sentimental adulation. Three centuries before the Christian Era, Menander said,

> "Of all wild beasts, on earth, or in the sea, the greatest is a woman."

In the same strain Virgil says,

> "Changeable and capricious always;"

and Livy,

> "Her mind affected by the meanest gifts;"

and Plautus,

> "Finding it much easier to do ill than well."

Juvenal says,

> "There's hardly a lawsuit but is caused by woman."

English literature abounds in quotations like the following from Otway:

> "What mighty ills have not been done by woman?
> Who was't betrayed the Capital? A woman!
> Who lost Marc Antony the world? A woman!
> Who was the cause of a long ten years' war, and
> Laid at last old Troy in ashes? Woman,
> Destructive, damnable, deceitful, woman!"

Or this from Granville:

> "Women from Eve have been the devil's tools.
> Heaven might have spared one torment when we fell,
> Not left us woman, or not threatened hell."

It is with a sense of relief that we turn to Addison and quote a different strain:

> "Heaven is in thy soul,
> Beauty and virtue shine forever round thee,
> Brightening each other! Thou art all divine."

Milton describes her thus:

> "Grace was in all her steps, heaven in her eye,
> In every gesture, dignity and love."

And Pope:

> "She moves a goddess and she looks a queen."

Look on this picture from Byron:

> "She walks in beauty like the night
> Of cloudless climes, and starry skies,
> And all that's best of dark and bright,
> Meet in her aspect and her eyes."

Longfellow says,

> "When she had passed, it seemed like the ceasing of exquisite music."

And Milton again,

> "O! fairest of creation, last and best
> Of all God's works, creature in whom excelled
> Whatever can to sight or thought be formed,
> Holy, divine, good, amiable, or sweet."

Through all the cynicism, satire, and exaggeration shown in these quotations, in all their apparent inconsistencies and absurdities, woman was viewed through a medium which the writer himself threw around her. It was he who clothed her with these attributes, and in his estimation she seems to have been regarded as a thing apart from himself—a being either so far below him that she was fit only for condemnation, or so far above him that he could only worship—never his exact equal or counterpart, endowed with the same faculties, prompted by the same motives, and incited by the same aspirations.

One of the strongest contrasts between barbarism, semi-barbarism, and the civilization of to-day, is shown in man's estimation of woman. In the barbaric state she was a beast of burden, or at best a slave. From the period of classic civilization until that of the Middle Ages, she was exalted by the chivalrous to the throne of a goddess; but in these modern days she has descended voluntarily from such an unsubstantial eminence, and seeks to be regarded only as man's equal, and enjoy all the rights and privileges that her faculties and endowments entitle her to. A generation since, the ultra-theorists of her sex began demanding a recognition of her so-called rights, and since then she has been knocking at the door of our industries, and asking that she may be permitted to become an independent and self-sustaining creature. To the credit of the other sex, it must be said that this desire has met with general sympathy and co-operation. Women have not been discouraged to any extent from entering any field of labor to which their tastes or necessities inclined them, until now there is hardly an occupation in which they may not be found. According to the census of 1880, there were half a million persons engaged in industrial occupations of all sorts in New York alone, of which nearly one third were women and girls. Women were then taking part in nearly every profession and every trade not requiring rough physical labor and exposure, to which manifestly they are not adapted. But the experience of this generation has been, in this particular, and still is, an experiment. Women have been undergoing the crucial test of proving their ability to do a man's work, like and equal to a man. The fruits of her ambition are beginning to develop. Like the pendulum of all revolutions when set in motion, it was likely to swing to the opposite extreme, and now true progress for woman seems to lie in less exacting and less pretentious fields.

There is no problem in sociology of greater importance now than this: In what way can a woman best become an independent and self-sustaining creature?

In no city of the world am I likely to find an audience more in sympathy with my subject than here in Boston. Boston, more than any other place I know, has the reputation of viewing a social problem without prejudice and free from bias. Her society has not been weakened by the traditions or example of aristocratic class distinctions, nor tainted by the aspiring aristocracy of parvenu wealth. London first, and New York second, of all cities are the most antagonistic foes to woman's social advancement, if she be self-supporting. In London, because the idea of gentility, derived from the aristocracy, makes all self-support, even among men, in a measure degrading. The gentlewoman in London may maintain her social position; a pauper, upon the charity of her friends; but earning her own living, never. In New York an aristocracy of wealth is growing which attempts to imitate that of birth, and its effects are equally disastrous.

It is no wonder that the young woman born of American parentage will not enter domestic service. From that moment she loses her individuality. No matter how light the service, no matter what physical comforts are provided, no matter how large the wages, there is no time which she can call her own; from early rising until time for bed, she is subject to the caprice and whims of a mistress whose orders are not always judicious nor always consistent. Domestic service in the rural districts, up to a recent period, could hardly be called service. It was rather the friendly help extended by the daughter of a neighbor of equal rank; and marked or real social distinction between mistress and maid was hardly thought of, for the families married and intermarried without a suspicion of class distinction. But domestic service to-day, and especially in towns and cities, involves all the apparent distinction which exists between mistress and slave. The servant loses her family name; she is Mary, or Katy, or Ellen; but Miss Brown or Miss Jones, never. The young woman of American birth who has received the best education of the public schools cannot enter domestic service without a feeling of degradation; and if she be of a higher grade of education, and fitted by nature and aspirations to adorn a higher station, what occupations are adapted to her?

Individuals of both sexes are by nature specially adapted to special employments. There are occupations which only the physical strength and endurance of the hardiest men are equal to, and others of quite as much importance are of so light a character that it would be considered effeminate in a man to engage in them. Some skilled labors belong to, and can only be prosecuted by, man by virtue of his superior strength. Occupations are suited to individuals, and not to a whole community, class or nationality. You would not make a beast of burden of a thorough-bred Arabian nor would you put the delicately reared and finely-cultured youth to carrying bricks and mortar. As civilization advances, there is created a wide range of employments that both sexes by nature are about equally adapted to; but occupations for women ought always to take into consideration the physical distinction of the sexes. In a state of refined civilization, woman is only man's equal mentally. If she were created originally as his physical equal, she has become enfeebled by long habits of dependence, and by the accumulated inheritance of weakening influences through countless generations.

John Stuart Mill maintains the absolute equality of the sexes in respect to psychical endowments and social capabilities, and goes so far in defence of his doctrine of the essential equality of the sexes as to say, "Bring women up like men, and they will be able to do everything that men do." But Dr. Hammond, in a recent publication, opposes this view, and makes her man's intellectual inferior in all except her emotional nature. Hammond says that the differences between the brain of an average male and the average female are "numerous and striking." "The shape is quite different. The convolutions of the male brain are more intricate; the sulci are deeper, the secondary fissures more numerous, and the specific gravity of both white and gray substance is greater in man than in woman. Difference in structure necessarily involves difference in function. Woman's brain is one from which emotion rather than intellect is evolved, and this circumstance, while it constitutes one of the strongest factors among those which are concerned in the happiness and preservation of the species, is at the same time one which thoroughly disqualifies her in whom it is manifested for those sterner duties which must be performed through the exercise of the intellectual faculties."

Her emotional nature is as much an inherent and irrepressible sexual quality as is her maternity, and in considering her fitness for any station cannot be ignored. In that very fact lies her special adaptability to certain conditions in life, and by the possession of that quality does she become unfitted for other callings. Her judgments are not the verdict of dilatory reason; she startles by the promptness of her decisions; but it is intuition rather than reason, and she is more likely to be influenced by sentiment than by logic. She is esthetical but not mathematical; imitative rather than inventive. In her lie the capabilities of the artist more than the mechanic. In exceptional cases she has reached the highest round in the ladder of fame in various branches of ideal art—in poetry, music, painting, and sculpture even, but she has not contributed to the utilitarian progress of the age by the invention of machines. "Who, however, will venture to say that the brain which evolves a mother's love, a wife's fidelity and self-abnegation, a sister's devotion, a woman's gentleness, forbearance and constancy, is not a better brain than one which prompts to making, executing and interpreting laws?" or to the planning of arctic voyages, the carrying of railways over mountains, to the building of suspension bridges, or the development of electrical science?

Woman's sphere is not in the advance—the scout and pioneer of an emigrant train, the sapper and miner in front of a besieging corps, nor the general in supreme command even. Her place is with the sick and wounded in the rear of the battle, where she is of more value to the cause she serves, by saving lives, than he who stands in front and kills an enemy.

Man may reach the highest capabilities of his intellect and his nature, independent and unaided by woman, but woman is not likely to reach the highest possibilities of her nature without the aid of man. Woman reaches her highest destiny when she becomes man's associate and helper, and her typical condition finds its best expression in the relation of man and wife. Longfellow, in "Hiawatha," puts it in this way:

"As unto the bow the cord is,
So unto the man is woman:
Though she bends him she obeys him;
Though she draws him yet she follows:
Useless each without the other."

But the question of marriage is not one over which she has entire control. Her innate modesty and refinement forbid her seeking a companion; she must wait and be sought. I do not believe this condition is a conventionalism of society intensified by long continuance; it must have always existed in some degree, and while woman possesses the qualities she now does, it always will.

But while she is waiting she must live.

The woman of to-day, who with self-respect and becoming dignity will be neither odalisque nor servant, asks herself the question, "What can I do?" "What am I by nature and education fitted for?"

It is not the working woman, as the term is generally applied, to which I now call your attention. It is not to the factory-girl, the shop-girl, nor to those who desire to be known as "sales-ladies." The shop-girl of to-day has been evovled to a large extent from the "cash-girl," and the sales-lady considers herself a higher evolution than the shop-girl; but each of these grades has received a preparatory training in the position which it had previously held, and in no case is superior education, culture or refinement, other than inborn refinement, of special value. An education equal to the position of cash-girl is a sufficient foundation upon which to base the training for the subsequent shop-girl or the later "sales-lady."

Your attention to-day is invited rather to a constantly enlarging class of refined and cultured women, who from a variety of causes are thrown upon their own resources, and obliged to support themselves.

What shall the woman do who, no longer young, has for the first half of her life learned to look nice and behave in a lady-like manner, whose education is the education of the upper middle class, when suddenly, through no fault of her own, or for the matter of that through her own fault, she is compelled to face the dismal problem of "how to live?" And if it be a serious question with the woman of middle age, it becomes tenfold more serious with the woman just entering womanhood. What are her chances in life outside the lottery of marriage, if her innate refinement and culture fit her for any station above that of mere working woman, into competition with whom she must inevitably come?

If any one question the number of such that are to be found in any community, let him put an advertisement in a city newspaper, offering genteel employment at a salary even ridiculously small, and he will be astonished at the army of women it will bring forth, of every age and nationality, and every social grade.

With the present liberal ideas, all the professions are open to her —law, theology, medicine, journalism, the lecture platform, and what not. It is only a question of natural and acquired fitness for either. In the first two, law and theology, we may safely say that up to the present time only exceptional women have made any progress, and even that is hardly such as to encourage others.

But medicine, of all the professions, is the one which can best utilize the special qualities which woman possesses. Medicine, as distinguished from surgery, is far from an exact science. The knowledge of cause and effect in medicine is still so uncertain that one is often inclined to believe them beyond the reach of reason. In fact, the best writers, the ablest reasoners, or the profoundest thinkers, as a rule, are not the best physicians. Disease and its cure frequently seem to defy all reason, and leave the charlatan with as long a list of successful cures as the scientist. The diagnosis of a disease would often be reached with as much certainty by intuition as it is now by reason. A consultation with an old country doctor, of limited education but large experience, will generally be of more value than the opinion of the most brilliant of recent graduates. While the former might not be able to formulate a reason for his opinion, the latter could overwhelm you with his.

Woman's sympathetic nature makes her eminently fitted for the care of the sick; and in the treatment of diseases of her own sex, and of all children, I cannot see why she should not be more successful than a man. I should not hesitate to trust her medicine, when I might beware of her surgery. Her inexactness would be fatal to her surgical operations, while her intuitions might be equal to the treatment of disease. But medicine involves years of application and study before a dollar can be earned, and to the young woman of otherwise liberal attainments, who must earn money now, it is a formidable undertaking.

Dentistry, as a specialty of medicine, has become almost a distinct profession—an ever-widening field, both in its acquirements and its achievements. Into it now are crowding hundreds of young men every year. This very increase of numbers is producing the effect of calling more attention to the subject, creating in the community a wider interest in the care and preservation of the teeth, and thus supply and demand are regulating each other. The necessity for the dentist is still far in advance of the supply. It is quite safe to say that if the entire community gave that attention to their teeth which is really needed for their health and comfort, there would be in the United States alone thirty thousand practitioners, instead of ten thousand, as at present.

Is there in dentistry a field for woman?

The experiment has already been tried to a limited extent. There are women now practicing dentistry who have gone through the curriculum of the dental colleges, passed the ordeal for graduation quite as successfully as their male associates, and set themselves up as independent practitioners; but the number is still very limited, and I doubt not they could be counted upon the fingers. The number is not large enough to prove woman's fitness or adaptability to the calling. Whatever success they may have attained may be accounted for by the fact that exceptional women can always be found—phenomenal, they might almost be called. For the present I should rather rank them with the exceptional few who have become in times past warriors and led troops to victory, who possessed more masculine qualities that feminine.

If my estimate of woman's characteristics be correct, then there is much in dentistry which is not within the scope of the average woman. I have said that woman is not inventive. Many of the processes in dentistry require that the inventive faculty should be largely developed. Woman is inexact. A majority of the operations on the natural teeth require mathematical precision, and to a very large extent the same operations require an excessive mental and physical strain, to which a woman is not physiologically equal. Like some other entire employments, there are things in dentistry to which a woman is manifestly not by nature adapted.

Practical dentistry is both a beneficent profession and a remunerative business, but it must be prosecuted as a business to insure a competency. In these days of millionaires and corresponding incomes, it is not unnatural that every one should desire to gratify his tastes in such ways as only wealth can command. A dentist ought to be a man of culture and refined tastes, and to surround himself with the products of luxury and refinement demands a larger income than one pair of hands can ordinarily earn. If he be one by natural endowments adapted to a profession, the influence of which becomes almost entirely personal, he will in time find a claim upon his services more than he can meet. In a crowded practice, the result of an attachment of patients for their dentist through half a life-time of service, there comes eventually the question, "How can two hands be made to do the work of four?" for this one pair of hands cannot be supplemented in the fond estimation of the patient by another pair of hands controlled by another brain. There is not a dentist in the land who could not perform a far greater number of operations than he now does if he could devote himself exclusively to such only as required his superior skill, and relegate all things of a minor character to an acceptable and competent assistant; nor does competent assistant here mean skilled service in the full sense of the term.

This is one of the most serious problems that the successful practitioner is called upon to solve; and the history of the efforts made to meet the difficulty by the employment of younger practitioners is a record, in many cases, of sad disaster. More than one man who has spent the best years of his life in the effort to establish an unblemished reputation, and to acquire an influential *clientele*, has in an unguarded moment placed his confidence and his patients in the care of some young man as an assistant, only to find his confidence met by base ingratitude, his practice divided and enticed from him, and his days embittered by the infamous scandals of his younger rival. The risk of such a result is always impending. In the very nature of the case, the young man must be recommended as capable of performing skillfully whatever operations are entrusted to him; and if he be of winning manners, he becomes then in the estimation of the patient the equal of the principal. The intimate relations that exist necessarily between operator and patient afford an opportunity for the assistant to make influence and capital for his future benefit as an independent practitioner. The temptation is rarely resisted, and it is almost impossible to prevent it by legal agreements, however binding. The day comes when the principal discovers, alas, too late, that the viper which he warmed into life has bitten its benefactor.

May not the profession turn to woman for the needed relief?

While I have said that certain skilled operations on the teeth, as now performed, are probably above the capacity of the average woman, I shall nevertheless maintain that there is a place for her in dentistry which can be better filled by her than in any other way. The possibilities of valuable assistance within her province are almost beyond enumeration. It is to the young woman of good breeding who has been favored with educational advantages, but who has no special artistic gift by nature, such as music or painting, which she can cultivate, that the opportunity opens to become a most useful adjunct to an honorable profession, and fill a demand in the great industrial hive for which she is eminently fitted.

But in what way can she be constantly and valuably employed?

She will save the time of the principal by meeting all calls and arranging appointments. She will keep the books of accounts and records, and write all but personal correspondence. She will have general care of the offices, and there will be a neatness and order in the arrangements and the instruments which a woman's good taste is sure to show. She stands at the side of the chair during an operation, and her ability to fill all the requirements of an assistant at that moment is unexcelled. As she becomes more familiar with the details of practice she will perform all the operations required upon the deciduous teeth, including fillings with any of the plastics. Her manner and her hand seem specially adapted to this class of practice, for children will more readily submit to necessary treatment at the hands of a gentle lady than to the same treatment by a man. She can go further, and take the entire charge of all regulating cases, with the occasional advice of the principal; and that branch of practice so dreaded by all, because of the apparent waste of time in the re-arrangement of splints, becomes in her hands a valuable source of income. In short, it is impossible to enumerate in detail the acquirements she will come to possess.

And all this is physiologically and psychologically within her power and scope, without unsexing herself, or in any wise destroying the charm which will always surround a refined woman.

There are a thousand and one little polite attentions to patients that good breeding require, but which unfortunately take the valuable time of the dentist, but which can be most graciously and acceptably performed by a young woman of pleasing manners and tact.

A woman's fidelity in such a position is in strong contrast with that of a man. She is not governed solely by self-interest; she is more faithful to her position than a man, irrespective of that interest. A man associated with you in business can rarely be depended upon longer than it is to his selfish interest to be so.

*The ideal assistant, in a dental office, is a woman of education and refinement, of pleasing manners and address, interested in her vocation, viewing it with pride and not with humiliation, and devoted to the welfare of all she is called upon to serve.*

The value of such an assistant can no more be measured in money than you can weigh with gold the value of the services of a sister of charity whose ministering care has brought the sick and suffering back into health.

One of the surest signs that a community approve of any departure in professional customs is the readiness with which quacks catch hold of a new idea, and advertise it for their own recognition and advancement. It is not an uncommon thing now to see in the columns of a metropolitan newspaper the advertisements of those who are holding out plausible inducements for patronage, in which it is stated that "a lady is in attendance."

I wish to make the position of "lady assistant" one which shall be recognized as no humble nor menial condition in any sense. It is not that of higher servant, nor an attendant, nor a "lady's companion" in social degree; but it is one of equal importance in its scope with that of the principal, and holds to him the same relation that the staff officer or leader of a brigade does to the general commanding.

If women are ever to become to any extent independent practitioners of dentistry, it is far better that they be an evolution of the assistant, where they have been trained in the business of practice. The few who would then desire to take a college course would be more likely to succeed. While I would not stimulate a woman's aspirations in this direction, I should not under the circumstances discourage her.

I cannot conceive a more virtuous, noble or exalted ambition in a young woman than that which dismisses marriage (for the sake of the support which it will bring) from her mind as the sole aim and object of her life, and addresses herself to the problem of an independent existence. If marriage is offered with affection, by one whom she can respect, esteem and love, she merges her independence in another, and life's highest possible attainments may be reached. But in the consideration of her own life-work, if marrying is ever regarded as anything but a remote possibility, her success in any permanent calling which she may undertake will be problematical.

Such a determination upon the part of any young woman shows a courage that the sterner sex can hardly appreciate. To deliberately relinquish all hope of attaining the consummation of earthly happiness, and resolutely mark out a work to which life's energies shall be devoted, requires a strength of purpose, of perseverance, of fortitude and of heroism, which proves her possessed of the noblest attributes a finite being can have. A man must be less than human who can witness the uncomplaining struggles of a woman, often for her very existence, without calling out all the tenderest sympathies which his nature possesses.

If it were but in my power, from henceforth not another woman in all the world should have one anxious thought for her physical or material welfare.

While in various parts of my discourse I may have seemed to belittle woman's powers and capabilities, I desire in closing to pay to the ideal representative of her sex the highest tribute that language can command. No man shall excel me in the homage I pay to woman because she is woman; to her sincere, unselfish and faithful devotion to her conception of duty; to her consistent and unwavering fidelity to her trusts; to the sympathy and consolation she brings to the suffering; to her bravery in adversity, her serenity in affliction, and to her benevolence, kindness and mercy, her trustfulness and constancy in all relations of life.

> "To blot from earth's vocabularies one
> Of all her names, were to blot out the sun."

